# Cognitive Judgment Bias Interacts with Risk Based Decision Making and Sensitivity to Dopaminergic Challenge in Male Rats

**DOI:** 10.3389/fnbeh.2016.00163

**Published:** 2016-08-23

**Authors:** Robert Drozd, Przemyslaw E. Cieslak, Michal Rychlik, Jan Rodriguez Parkitna, Rafal Rygula

**Affiliations:** ^1^Affective Cognitive Neuroscience Laboratory, Department of Behavioral Neuroscience and Drug Development, Institute of Pharmacology Polish Academy of SciencesKrakow, Poland; ^2^Laboratory of Transgenic Models, Department of Molecular Neuropharmacology, Institute of Pharmacology Polish Academy of SciencesKrakow, Poland

**Keywords:** rat, probability discounting, ambiguous-cue interpretation, risk, pessimism, optimism, cognitive judgment bias, dopamine

## Abstract

Although the cognitive theory has implicated judgment bias in various psychopathologies, its role in decision making under risk remains relatively unexplored. In the present study, we assessed the effects of cognitive judgment bias on risky choices in rats. First, we trained and tested the animals on the rat version of the probability-discounting (PD) task. During discrete trials, the rats chose between two levers; a press on the “small/certain” lever always resulted in the delivery of one reward pellet, whereas a press on the “large/risky” lever resulted in the delivery of four pellets. However, the probability of receiving a reward from the “large/risky” lever gradually decreased over the four trial blocks. Subsequently, the rats were re-trained and evaluated on a series of ambiguous-cue interpretation (ACI) tests, which permitted their classification according to the display of “optimistic” or “pessimistic” traits. Because dopamine (DA) has been implicated in both: risky choices and optimism, in the last experiment, we compared the reactivity of the dopaminergic system in the “optimistic” and “pessimistic” animals using the apomorphine (APO; 2 mg/kg s.c.) sensitivity test. We demonstrated that as risk increased, the proportion of risky lever choices decreased significantly slower in “optimists” compared with “pessimists” and that these differences between the two groups of rats were associated with different levels of dopaminergic system reactivity. Our findings suggest that cognitive judgment bias, risky decision-making and DA are linked, and they provide a foundation for further investigation of the behavioral traits and cognitive processes that influence risky choices in animal models.

## Introduction

Since Chevelier de Mere (1607–1684) asked Blaise Pascal for help with identifying a mathematical answer for why he consistently lost money playing a certain game of dice, intellectuals, economists and psychologists have studied how different aspects of human cognition influence risk-related decision making. Recently, cognitive biases and distortions, such as unrealistic optimism and/or pessimism, have been proposed to play crucial roles in risky decision making and to cause the establishment and maintenance of mental disorders such as pathological gambling.

The scientific definitions of optimism and pessimism focus on expectations for the future (Carver and Scheier, 2014). An individual who expects eventual success is considered optimistic, whereas an individual who expects failure is considered pessimistic (Carver and Scheier, 2014). The rather simple difference between anticipating good vs. anticipating bad is linked to core processes that underlie behavior. Optimistic and pessimistic people differ in how they confront problems and in how well they cope with adversity, and these differences substantially impact their lives. The psychological literature has provided a growing body of evidence that optimism is a highly advantageous trait that is linked to enhanced performance in the domains of academics (Chang and Bridewell, 1998; Montgomery et al., 2003), athletics (Gould et al., 2002; Gordon, 2008), health (Scheier and Carver, 1987; Brydon et al., 2009) and work (Seligman and Schulman, 1986; Kluemper et al., 2009). Optimists have also been shown to engage more in treatment programs (e.g., psychotherapy, nutrition and education), and consistent with their greater engagement in other high-priority tasks (Geers et al., 2009; Carver and Scheier, 2014), optimistic individuals are known to work harder at maintaining their relationships (Segerstrom, 2007; Rand, 2009).

Although the benefits of optimism are clear, over-optimistic expectations can sometimes have negative consequences and lead to poor performance. In certain situations, optimism has been hypothesized to be detrimental (Weinstein, 1982, 1987; Tennen and Affleck, 1987; Weinstein et al., 2005). For example, risky behavior may be more common among optimists than among pessimists, and optimism may diminish the likelihood that an individual will take preventive steps to avoid an adverse outcome. In fact, several studies have revealed that optimistic individuals tend to underestimate potential obstacles and threats, to take risks, and to persist in investing in hopeless endeavors (Felton et al., 2003; Trevelyan, 2008; Hmieleski and Baron, 2009). Optimists are also more likely than pessimists to take risks and continue gambling after losing money (Gibson and Sanbonmatsu, 2004). Despite these reports, information about the relationship between optimism and risky decision making is still scarce and thus requires a systematic investigation.

In the present study, we used two recently developed animal paradigms, the probability discounting (PD) test and the ambiguous-cue interpretation (ACI) test, to evaluate differences in the approach to risk-taking between animals displaying “optimistic” and “pessimistic” traits. Because the dopamine (DA) system is thought to play an important modulatory role in both: risky choices and optimism (St Onge and Floresco, 2009; Sharot et al., 2012), we also evaluated the reactivity of the DA system in individual animals using the apomorphine (APO) sensitivity test.

To establish individual differences in risky decision making, we initially trained and tested the rats on the operant version of the PD task (St Onge and Floresco, 2009). In this behavioral paradigm, over discrete trials, animals choose between two levers; a press on the “certain/small” lever always results in the delivery of one reward pellet, whereas a press on the “risky/large” lever results in the delivery of four pellets. However, the probability of receiving a reward from the “large/risky” lever decreases across the four trial blocks (100, 50, 25 and 12.5%). The lever press response pattern across the four blocks is used as an indicator of the rat’s attitude towards risk; a choice bias towards the larger, probabilistic reward indicates risk seeking, while a preference for the small/certain reward indicates risk aversion (Floresco et al., 2008; St Onge and Floresco, 2009).

To determine each rat’s individual level of “optimism”/“pessimism”, we subjected the animals to a number of consecutive ACI tests (Harding et al., 2004; Enkel et al., 2010; Rygula et al., 2012) that were conducted at 1-week intervals. In the ACI paradigm, rats are trained to press a lever in an operant conditioning chamber to receive a food reward when they hear a specific tone and to press another lever in response to a different tone to avoid punishment by an electrical foot shock. The tones, which serve as discriminative stimuli, acquire positive and negative valence, and the training continues until the rats demonstrate stable, correct discrimination. After the animals attain stable discrimination performance, they are then tested. The ambiguous-cue testing consists of a discrimination task, as described above, but with the presentation of additional tones with intermediate frequencies that are between the positive and negative tones. The lever press response pattern to the ambiguous cues is considered an indicator of the rat’s expectation of a positive or negative event; in other words, it is a measure of “optimism” or “pessimism”, respectively (for details, see Enkel et al., 2010; Rygula et al., 2012, 2013; Papciak et al., 2013). Based on the results of this cognitive bias screening (CBS), we classified individual animals into one of two groups: those displaying a positive judgment bias, which were referred to as “optimistic”, and those displaying a negative judgment bias, which were referred to as “pessimistic”.

To evaluate the reactivity of the DA system in individual animals the rats were subjected to the APO sensitivity test. In this test, rats injected with the DA receptor agonist APO display different types of stereotyped oral behavior (e.g., gnawing, licking and sniffing) that indicate different degrees of DA system stimulation. Stereotyped sniffing behavior changes to licking behavior and finally to gnawing behavior with increasing DA system reactivity.

We hypothesized that “optimism” and “pessimism” would be associated with differences in the willingness to take a risk in the PD task and predicted that these differences would be correlated with the reactivity of the DA system in individual animals.

## Materials and Methods

### Ethics Statement

All experiments were conducted in accordance with the European Union guidelines for the care and use of laboratory animals (2010/63/EU) and were reviewed and approved by the local Bioethics Committee. The authors attest that all efforts were made to minimize the number of animals used and their suffering.

### Subjects and Housing

We used 40 male Sprague Dawley rats (Charles River, Germany), weighing between 175 and 200 g upon arrival. We housed the rats in groups (4 animals/cage) in a temperature (21 ± 1°C) and humidity (40–50%) controlled room. The rats were kept under a 12/12-h light/dark (L/D) cycle (lights on at 07:00 h). The animals were mildly food restricted to approximately 85% of their free-feeding weights what was achieved by providing 15–20 g of food per rat per day (standard laboratory chow, Labofeed, Kcynia, Poland). Food was restricted beginning at 1 week prior to training. Water was freely available, with the exception of during the test sessions. All the behavioral procedures were performed during the light phase of the L/D cycle.

### Apparatus

The operant tests were performed in 16 computer-controlled boxes for operant-conditioning (Med Associates, St Albans, VT, USA); each box was equipped with a speaker, a light, a liquid dispenser (set to deliver 0.1 ml of 5% sucrose solution (ACI task), a pellet dispenser (set to deliver 45 mg pellets; Bioserv, Frenchtown, NJ, USA (PD task)), a grid floor through which scrambled electrical shocks (0.5 mA (ACI task)) could be delivered, and two retractable levers. The levers were located on opposite sides of the feeder. All of the behavioral protocols, including the data acquisition and recordings, were programmed in Med State notation code (Med Associates). The experimental procedures for the PD test were modified from the procedures described by St Onge and Floresco (2009). The experimental procedures for the ACI test used in this study were performed according to Enkel et al. (2010) with modifications, and they have been described in detail elsewhere (Rygula et al., 2012, 2015).

### Behavioral Training and Testing in the Probability Discounting Task

The training and testing protocols used have been previously described elsewhere (Floresco et al., 2008; St Onge and Floresco, 2009). Briefly, the rats were first trained to press both levers under a fixed-ratio schedule (1:1) to a criterion of 60 presses in 30 min. Half of the animals were trained first on the left lever and later on the right lever, and the other half were trained in the reverse order. Subsequently, we trained the rats on a simplified version of the full task. Each training session began in darkness, with the levers retracted. Each trial began with the illumination of the house lights and extension of one of the two levers into the operant box. If the animal responded within 10 s, the lever retracted and a single sucrose pellet (45 mg, Bio-Serv, Frenchtown, NJ, USA) was delivered with 50% probability. If the animal did not respond to the lever within 10 s, the lever retracted, the house light was switched off, and the trial was scored as an omission. The trials lasted 40 s each and the training session consisted of 90 trials. We used this procedure to familiarize the animals with the probabilistic nature of the full task. The training took approximately 4–5 days (to a criterion of 80 or more successful trials).

**Figure 1:**
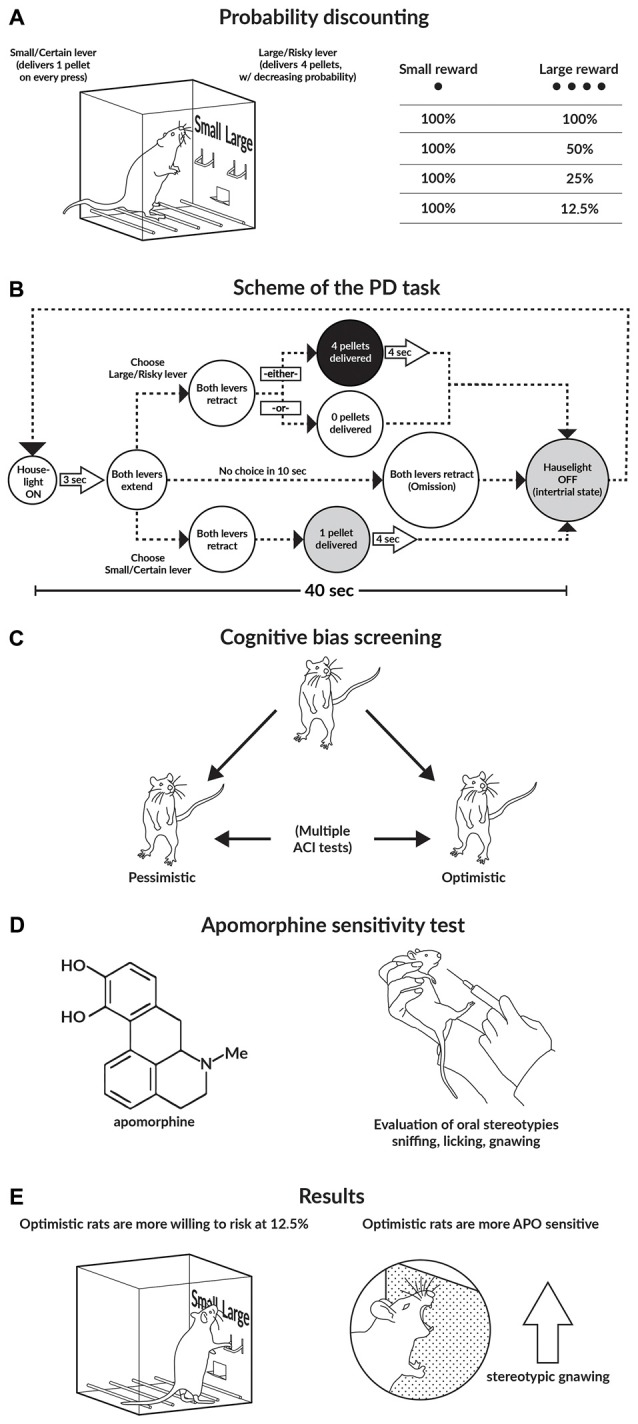
**Graphical abstract of the study. (A,B)** Schematics of the probability discounting (PD) task. **(C)** Schematic of the cognitive bias screening (CBS) procedure. To assess cognitive judgment bias as a trait, we examined the animals in a series of 10 consecutive ambiguous-cue interpretation (ACI) tests conducted at 1-week intervals. Based on the average cognitive bias index obtained from these 10 ACI tests, the rats were divided into 2 subgroups: “optimistic” and “pessimistic”. **(D)** Apomorphine (APO) sensitivity test. After the animals were injected with APO (2 mg/kg), they were placed into an open field for 1 h. All of the stereotyped behaviors displayed by the animals (sniffing, licking, and gnawing), as well as their general locomotor activity, were recorded. **(E)** Main results of the study.

During PD testing, the rats received daily sessions of 72 trials that were separated into four blocks of 18 trials. The entire session took 48 min to complete, and the animals were trained 5 days per week. At the beginning of each session the levers were retracted and the house light was switched off (the intertrial state). Each trial began with the illumination of the house light and extension of one or both levers (the schedule of a single trial is shown in Figure 1B). One lever was designated as the small/certain lever and the other was designated as the large/risky lever; these designations were counterbalanced left/right and remained consistent throughout the training phase. When a lever was chosen, both levers were retracted. Selection of the small/certain lever always resulted in the delivery of one sucrose pellet; however, selection of the large/risky lever resulted in the delivery of four sucrose pellets with a particular probability (see below). Multiple sucrose pellets were delivered 0.5 s apart. If the animal failed to press a lever within 10 s, the operant conditioning box was reset to the intertrial state until the next trial and scored as an omission. Each of the four blocks began with eight forced choice one-lever trials (four trials randomized in pairs for each lever), which allowed the animals to learn the probability of receiving reinforcement during each block and the amount of food associated with each lever press. Next, there were 10 free-choice 2 lever trials, and the rats chose either the large/risky or the small/certain lever. The probability of obtaining four sucrose pellets after pressing the large/risky lever successively decreased across the blocks; the probabilities averaged 100% in block 1, 50% in block 2, 25% in block 3, and 12.5% in block 4. For each session and trial block, the probability of receiving the large reward was drawn from a set probability distribution. Therefore, on any given day, the probabilities in each block could vary, but on average across many training days, the actual probability experienced by the rat approximated the set value. Using these probabilities, selection of the large/risky lever was advantageous in the first two blocks, balanced during the 25% block and disadvantageous in the last block.

The rats were trained on the task until the following criteria were met by the group: (1) In at least 80% of the trials they chose the large/risky lever during the first trial block (100% probability); (2) In less than 60% of the trials they chose the large/risky lever during the final trial block (12.5% probability); and (3) the consistent choice patterns fulfilling criteria 1 and 2 were demonstrated on three consecutive days. Individual patterns of PD behavior over the three criterion days were used to compare risk-taking behavior between the rats displaying “optimistic” and “pessimistic” traits.

### Behavioral Parameters Measured in the Probability Discounting Test

The primary dependent measure of interest was the proportion of choices directed towards the large/risky lever during each block of free-choice trials, taking into account trial omissions. For each block, this proportion was calculated by dividing the number of choices of the large/risky lever by the total number of successful trials.

### Behavioral Training for the Ambiguous-Cue Interpretation Test

After training and testing on the PD task, the rats were re-trained and re-tested on the ACI paradigm. As mentioned previously, the experimental training and testing procedures for the ACI paradigm used in this study have been described previously (Enkel et al., 2010; Rygula et al., 2012, 2015).

Briefly, the animals were initially trained to press one lever when a “positive” tone (2000 Hz at 75 dB, counterbalanced) signaled reward (20% sucrose solution) availability and to press a second lever when another “negative” tone (9000 Hz at 75 dB, counterbalanced) signaled a forthcoming punishment (electric foot shock: 0.5 mA, 10-s). By pressing the appropriate levers, the rats could either avoid the punishment or receive a reward. 10 s intertrial intervals (ITIs) separated the tone presentations, and training sessions lasted for 30 min. The rats had to reach the criteria of at least 90% correct responses to the tone signaling reward availability over three consecutive training sessions and at least 60% correct punishment-prevention responses over three consecutive training sessions to proceed to discrimination training.

During the discrimination-training phase, the animals were trained to discriminate between 20 negative and 20 positive tones presented in pseudorandom order, by pressing the appropriate levers (as learned in the previous training stage) to maximize reward delivery and minimize punishment. Training sessions lasted 40 min. The animals were required to do a minimum of 70% correct responses for each lever over three consecutive discrimination sessions to qualify for the ACI testing.

### Ambiguous-Cue Testing

During the ACI testing sessions, the rats were exposed to 20 positive, 20 negative and 10 ambiguous (5000 Hz at 75 dB) tone presentations. Each test session lasted for 50 min. The tones were played in a pseudo-randomized order and were separated by 10-s ITIs. The responses to each tone (positive, ambiguous and negative) presented during the ACI testing were analyzed as a proportion of the overall number of responses to a given tone. To calculate the cognitive bias index, we subtracted the proportion of negative responses to the ambiguous cues from the proportion of positive responses to the ambiguous cues, resulting in values of between −1 and 1. Values of greater than or equal to 0 indicated an overall positive judgment and “optimistic” interpretation of the ambiguous cue, whereas values of below 0 indicated an overall negative judgment and “pessimism”.

### Cognitive Bias Screening

The CBS has been described in detail in Rygula et al. (2013). Briefly, to assess the “optimistic” and “pessimistic” traits, we subjected the rats to a series of ten consecutive ACI tests, conducted at 1-week intervals. Based on the average values of cognitive bias index data obtained from these 10 ACI tests, the animals were divided into two experimental groups: “optimistic” and “pessimistic”.

### Apomorphine Sensitivity Test

The APO sensitivity test that was applied in our study was modified from the procedures previously described by Havemann et al. (1986) and Surmann and Havemann-Reinecke (1995). Briefly, immediately after the animals were injected with APO (2 mg/kg s.c.), they were placed in an open field (66 × 56 × 30 cm) for 1 h. Next, scoring was performed in 30-s periods with 5-min intervals. Within each scoring period, we recorded all of the stereotyped behaviors displayed by the animals. Each 30-s scoring period was classified according to the stereotyped behavior exhibited during the longest period (e.g., when gnawing, licking and sniffing lasted for 20, 7 and 3 s, respectively, the scoring period was classified as “gnawing”). When two stereotyped behaviors were displayed for equal amounts of time (e.g., 15 s each), we scored both behaviors. Stereotyped sniffing was defined as rhythmic movement of the snout and head along the cage wall or floor, accompanied by rapid vibrissae movements. Stereotyped licking was characterized by protrusion of the tongue against the cage floor or wall, and stereotyped gnawing was scored when the animal gripped the cage wall or floor between its teeth. We also scored stereotyped climbing, characterized by vertical scratching and rhythmic, alternating movements of both forelegs on the cage wall (Havemann et al., 1986).

### Experimental Design

The experimental design and schematic representations of the procedures are presented in Figures 1A–D. The animals that met the criteria and successfully completed the training were tested on the PD task three times over three consecutive days and were then re-trained on the ACI task. Each animal was subjected to the CBS procedure as previously described. After the rats were evaluated to determine their degree of “optimism” or “pessimism”, they were divided into “optimistic” and “pessimistic” experimental groups. To assess whether the traits of “pessimism” and “optimism” interacted with risky behaviors, we compared the average performances of the “optimistic” and “pessimistic” animals on the three PD tests.

Notably, the PD testing preceded the ACI testing because the levers remained unbiased during the second test, only if the tests were conducted in that order. If the animals had been tested first on the ACI paradigm, then the levers would have acquired negative and positive valences via their associations with a punishment or palatable reward, and they would not have been able to be used as reliable operands in the PD paradigm.

Because DA has been implicated in both: risky choices and optimism, in the final experiment, we compared the reactivity of the DA system between the “optimistic” and “pessimistic” animals using the APO sensitivity test.

### Statistics

The data were analyzed using SPSS (version 21.0, SPSS Inc., Chicago, IL, USA). The Kolmogorov-Smirnov test was used to assess the distribution of the cognitive bias index data. Differences in PD between the “optimists” and “pessimists” were analyzed by 2-way analysis of variance (ANOVA), with the cognitive bias index as a between-subject factor (2 levels: “optimism” and “pessimism”) and the trial block as a within-subject factor (4 levels: 100, 50, 25 and 12.5%).

The differences in the frequency of “optimism” and the speed and distance traveled following APO administration between the “optimists” and “pessimists” were analyzed using *t*-tests. Differences in the processing of the positive and negative tones and ambiguous cues between the “optimistic” and “pessimistic” animals were investigated by 4-way ANOVA, with cognitive bias as the between-subject factor (2 levels: optimistic and pessimistic) and lever (2 levels: positive and negative), tone (3 levels: positive, ambiguous and negative) and test (10 levels: baseline test 1–10) as the within-subjects factors.

Differences in the length of training for the ACI test and insensitivity to the APO challenge (number of different stereotyped behaviors) between the “optimists” and “pessimists” were analyzed separately using the Mann-Whitney test because the data were not normally distributed. Finally, the Spearman correlation coefficient between the cognitive bias index and the frequency of gnawing was determined. For pair-wise comparisons, the values were adjusted using Sidak’s correction factor for multiple comparisons (Howell, 1997). All of the tests of significance were performed at α = 0.05. Homogeneity of variance was confirmed using a Levene’s test. For repeated-measures analyses, sphericity was also verified using Mauchly’s test. The data are presented as the mean ± SEM.

## Results

### Probability Discounting

**Figure 2:**
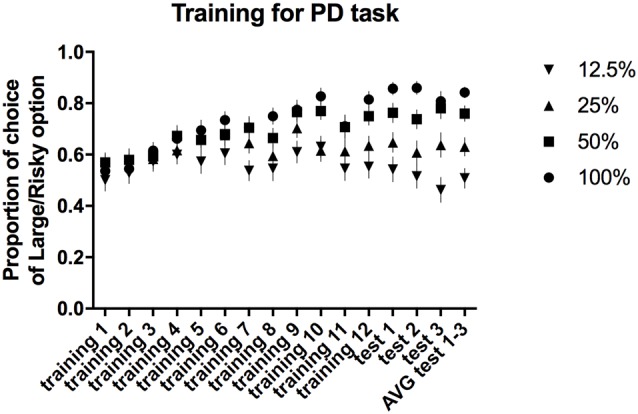
**PD training and testing.** The average proportion of times the large/risky lever was selected across the four trial blocks (100, 50, 25 and 12.5%) during the training and test sessions.

All of the trained animals reached the criteria for stable PD as a group (defined as a selection of the large/risky lever during the first trial block (100% probability) in at least 80% of trials and selection of the large/risky lever during the last trial block (12.5% probability) in less than 60% of trials, maintained over three consecutive days) after 15 sessions. The average proportion of risky lever choices made by all of the rats in each block of free-choice trials during the 12 training and three test sessions is presented in Figure 2. Repeated-measures ANOVA of the average choice data from the three PD tests revealed a significant main effect of trial block (*F*_(3,84)_ = 30.55, *p* ≤ 0.001). *Post hoc* multiple comparisons revealed significant differences in the proportion of choices of the large/risky lever between the 100% vs. 25%, 100% vs. 12.5%, 50% vs. 12.5%, and 25% vs. 12.5% trial blocks (*p* ≤ 0.001), as well as between the 50% vs. 25% trial blocks (*p ≤ 0.01)*.

### Ambiguous-Cue Testing

To compare the risk-taking behaviors between the “optimistic” and “pessimistic” animals, after the PD testing, the rats were trained for the ACI paradigm and on the basis of the CBS procedure, classified as “optimistic” or “pessimistic”. Only 30 out of 40 trained rats, reached the criteria and qualified for the CBS. The animals that were further classified as “optimistic” reached the criteria of the positive tone, negative tone and discrimination training after 4.8 ± 0.3, 30.1 ± 5.7, and 60.1 ± 8.2 days, respectively, whereas those classified as “pessimistic” reached the same criteria after 4.2 ± 0.17, 32.1 ± 3.7 and 49.7 ± 5.7 days, respectively. The Mann-Whitney test revealed that the total length of training did not significantly differ between the “pessimistic” and “optimistic” animals (medians = 74 and 94, respectively; *U* = 83, NS).

The average cognitive bias index of all of the tested rats, which was determined based on CBS, was 0.09 ± 0.06. The cognitive bias index data collected during the CBS were normally distributed (*Z* = 0.12, *N* = 30, Kolmogorov-Smirnov test).

Analysis of the animals’ responses to the negative and positive levers following presentation of the reference and ambiguous tones across the CBS indicated the absence of test-retest effects. Despite the significant lever × test × tone interaction (*F*_(18,504)_ = 2.07, *p* < 0.01), *post hoc* pair-wise comparisons revealed the absence of unequivocal patterns in between-test differences.

### “Optimistic” vs. “Pessimistic” Animals

**Figure 3:**
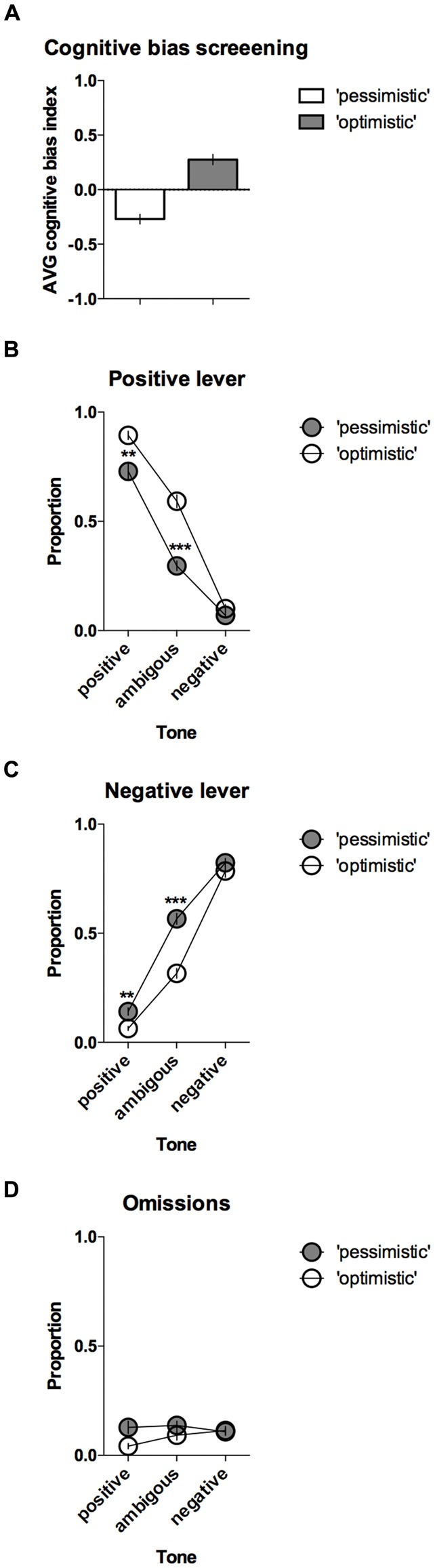
**“Optimistic” vs. “pessimistic” animals; results of the CBS. (A)** The mean ± SEM of the cognitive bias index of the animals classified (based on 10 ACI tests) as “pessimistic” (filled bar, *N* = 20) vs. “optimistic” (open bar, *N* = 10). A cognitive bias index of above 0 indicates an overall positive judgment and “optimistic” interpretation of the ambiguous cue. **(B)** The mean ± SEM proportions of positive, **(C)** Negative and **(D)** Omitted responses to the trained and ambiguous tones in the “pessimistic” (filled circles, *N* = 20) and “optimistic” (open circles, *N* = 10) rat groups. ** and *** indicate significant (*p* < 0.01 and *p* < 0.001, respectively) differences between the “optimistic” and “pessimistic” animals.

The CBS results enabled the animals to be separated into two groups, “pessimistic” (*N* = 20, average (AVG) cognitive bias index <0) and “optimistic” (*N* = 10, AVG cognitive bias index > 0), that differed significantly in their interpretation of the ambiguous cues over time (Figure 3A). The “pessimistic” animals had an average cognitive bias index, ranging from −0.02 to −0.80, whereas the “optimists” displayed cognitive bias index in the range from 0.00 to 0.48.

Further analysis revealed significant differences in the response patterns of the “pessimistic” and “optimistic” groups (there was a significant lever × tone × cognitive bias interaction (*F*_(2,56)_ = 14.40, *p* < 0.001)). In response to the ambiguous tone, the “optimistic” animals responded significantly more often (*p* < 0.001) to the positive lever (Figure 3B) and significantly (*p* < 0.001) less often to the negative lever (Figure 3C) than their “pessimistic” counterparts. The “optimistic” animals also responded significantly (*p* < 0.01) more often to the positive lever in response to the reference positive tone (Figure 3B) and significantly (*p* < 0.01) less often to the negative lever in response to the reference negative tone (Figure 3C). Analysis of the trials in which the animals did not respond (omissions) revealed a significant cognitive bias × tone interaction (*p* = 0.018) and a trend (*p* = 0.051, Sidak *post hoc* test) towards a higher proportion of omitted trials in the “pessimistic” animals compared to the “optimistic” animals following presentation of the positive reference tone (Figure 3D).

**Figure 4:**
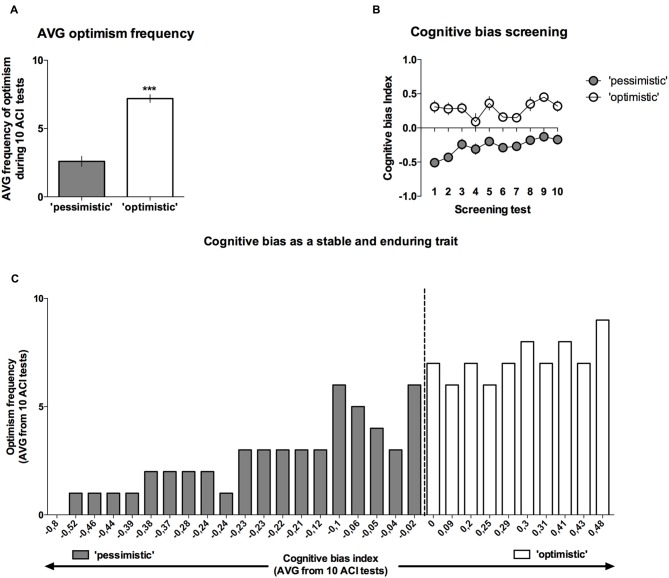
**Cognitive bias as a stable and long-lasting behavioral trait.** Out of 40 trained rats, only 30 reached the criterion and qualified for the CBS. **(A)** The mean ± SEM of the “optimism frequency” (number of ACI tests out of the 10 ACI tests that comprised the CBS that resulted in a cognitive bias index above “0”) of the animals that were classified as “optimistic” (open bars, *N* = 10) and “pessimistic” (filled bars, *N* = 20). **(B)** The mean ± SEM of the cognitive bias index of the animals that were classified as “optimistic” (open circles, *N* = 10) and “pessimistic” (filled circles, *N* = 20) across all 10 baseline ACI tests. **(C)** Histogram of the “optimism” frequency in relation to the valence of the individual cognitive bias index (AVG from CBS) in all (*N* = 30) animals. ***Indicates a significant (*p* < 0.001) difference between the “optimistic” and “pessimistic” animals.

Analysis of the “optimism” frequency (number of tests in which the cognitive bias index of an individual animal was greater than zero, out of the 10 CBS sessions) revealed that on average, the rats classified as “pessimists” were significantly (*p* < 0.001) less frequently “optimistic” than their “optimistic” conspecifics (*t*_(28)_ = 8.09, Figures 4A,C).

As mentioned previously, although the cognitive judgment bias index fluctuated in both groups of animals (there was a significant lever × test × tone interaction (*F*_(18,504)_ = 2.07, *p* < 0.01)), the differences between the “pessimistic” and “optimistic” remained constant across the screening period (there was no significant cognitive bias × test interaction; *F*_(9,252)_ = 1.38, NS), indicating that the traits were stable (Figure 4B).

### Interrelation Between Cognitive Judgement Bias and Probability Discounting

**Figure 5:**
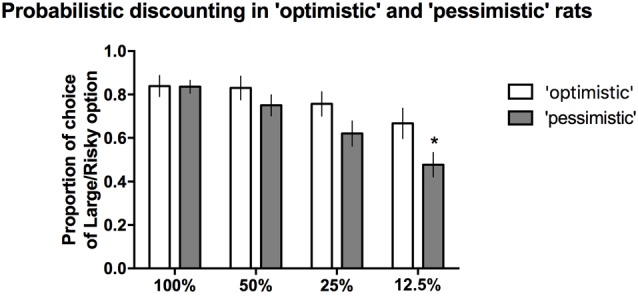
**PD in the “pessimistic” and “optimistic” rats.** The average proportion of times the large/risky lever was selected by the “optimistic” (*N* = 10, open bars) and “pessimistic” (*N* = 20, filled bars) animals across the four trial blocks (100, 50, 25 and 12.5%) of the three PD test sessions. The “optimists” were significantly (*p < 0.05) more likely than the “pessimists” to choose the large/risky lever when the probability of receiving a large reward was the lowest (12.5%). The data are presented as the mean ± SEM.

When the probability of reward was high (100%, 50%) or intermediate (25%), the proportion of time that the large/risky lever was chosen did not differ between the “optimists” and “pessimists” (Figure 5). However, compared to “pessimism”, “optimism” was associated with a significantly (*p ≤ 0.05) higher proportion of selection of the large/risky lever in the last block, when the probability of receiving a reward was the lowest (12.5%, Figure 5). Two-way repeated-measures ANOVA of the average choice data from the 3 PD tests revealed a significant cognitive bias × trial block interaction (*F*_(3,84)_ = 3.53, *p* ≤ 0.05).

### Interrelation Between Cognitive Judgement Bias and Sensitivity to Dopaminergic Stimulation in the Apomorphine Test

**Figure 6:**
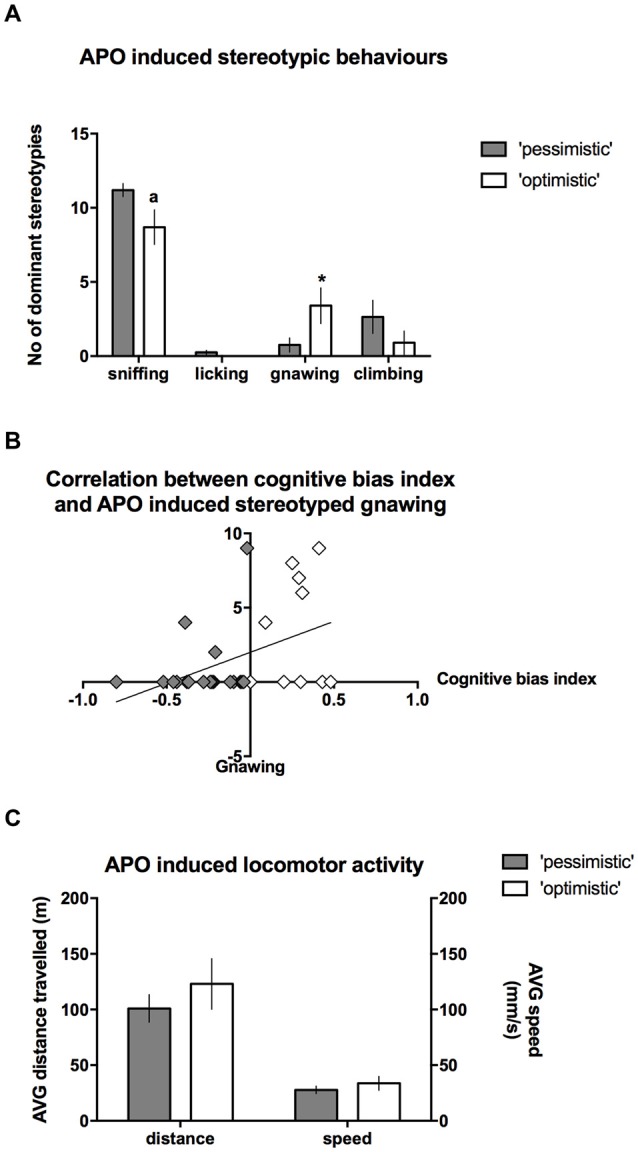
**Stereotyped behaviors in the “optimistic” and “pessimistic” animals following APO challenge. (A)** The number (mean ± SEM) of dominant stereotyped behaviors scored during 30-s scoring blocks throughout a 1-h period after administration of 2 mg/kg APO to “optimistic” (open bars, *N* = 10) and “pessimistic” (filled bars, *N* = 20) animals. **(B)** The correlation between the cognitive bias index and APO-induced stereotyped gnawing. **(C)** The AVG speed of movement (mm/s) and distance (m) traveled in the open field by the animals classified as “optimistic” (*N* = 10, open bars) and “pessimistic” (*N* = 20, filled bars) during a 1-h period following APO (2 mg/kg s.c.) administration. The data are presented as the mean ± SEM. *Indicates a significant (*p* < 0.05) difference between the “optimistic” and “pessimistic” animals. “a” Indicates statistically significant trend (*p* = 0.088) towards difference between the “optimistic” and ”pessimistic” animals.

After the administration of APO, the intensity of stereotyped climbing (Mann-Whitney *U* = 82) and licking behaviors (Mann-Whitney *U* = 85) did not differ between the “optimists” and “pessimists”; however, “optimism” was associated with significantly (Mann-Whitney *U* = 62.5, *p* ≤ 0.05) more intense gnawing behavior (Figure 6A) and a statistical trend (Mann-Whitney *U* = 66.5, *p* = 0.088) towards less intense sniffing (Figure 6A). Correlation analysis revealed that the cognitive bias index was significantly positively correlated (*r* = 0.39, *N* = 30, *p* < 0.05) with the frequency of stereotyped gnawing (Figure 6B). We observed no significant differences in APO-induced locomotor activity, as expressed by the speed of locomotion or the distance traveled in the open field (*t*_(28)_ = 0.93 and 0.91, respectively, NS, Figure 6C).

## Discussion

In the current study, we used two recently developed behavioral paradigms to investigate how “optimistic” and “pessimistic” traits determine animals’ approaches to risky decision making. The results of our study demonstrated that in rats, “optimistic’ judgment bias was associated with an increased propensity to make a risky choice, but only when the level of risk was high and not when it was low or intermediate. ‘Optimism” was also associated with increased sensitivity of the DA system, as indicated by the increase in stereotyped gnawing following APO challenge (Figure 1E).

To the best of our knowledge, this is the first study to systematically assess the effects of cognitive judgment bias on risk-based decision making, and the results indicate that “optimistic” animals display more risky choices.

The interrelations between cognitive judgment bias and other forms of decision making and associated cognitive processes have been previously studied in our laboratory. In a recent study, we examined whether the traits of “optimism” and “pessimism” are associated with differences in the interpretation of gambling outcomes in the rat slot machine task (Rafa et al., 2015). We demonstrated that although the “pessimistic” and “optimistic” animals did not differ in their propensity to interpret the “clear win” (light pattern 3 × ON), “near miss” (light pattern 2 × ON) and “near loss” (light pattern 1 × ON) outcomes as “win” trials, these animals differed significantly in their interpretation of the “clear loss” (light pattern 3 × OFF) outcome. The “pessimists” were significantly less likely than the “optimists” to press the “collect” lever following the “clear loss” outcome, suggesting that the “pessimists” interpreted the clearly hopeless situation more negatively. The results of the present study are consistent with these previous findings, in which differences between “optimists” and “pessimists” appeared only in a “hopeless” situation, when the risk was greater. The cognitive judgment bias appears to influence animals’ decisions primarily in extreme situations. Because the degree of ambiguity has been proposed to influence the perception of likelihood or risk, one plausible explanation for this phenomenon is that in the PD task used in our study, at lower levels of risk, the “optimistic” and “pessimistic” animals both recognized the probability and adjusted their decisions accordingly. However, at the highest level of risk, the probability was ambiguous, and the “optimists” adjusted their expectations more positively than the “pessimists”. A similar mechanism could have also determined the choices of the animals in the aforementioned study that compared the decision making of “optimists” and “pessimists” in the rat slot machine task. Einhorn and Hogarth (1986) have argued that when individuals are given a probability that they believe is ambiguous, they use the probability as an anchor and adjust it upwards or downwards, and one important factor affecting this adjustment is whether the individuals are pessimistic or optimistic about the outcome.

In another study conducted recently in our laboratory (Rygula et al., 2015), we have demonstrated that animals displaying the “optimistic” trait are more motivated to obtain a sweet sucrose reward than their “pessimistic” conspecifics. These findings could provide another plausible explanation for the effects observed in the present experiments. As the expectancy-value theory posits that an individual’s expectations of success and the subjective value that they place on succeeding coincide with their motivation to perform different achievement tasks (Wigfield and Eccles, 2000), the “optimistic” animals were probably more motivated to obtain a risky reward than the “pessimistic” ones. Indeed, as postulated by Carver and Scheier (2014), when confronting a challenge (e.g., a high-risk situation), optimists are more persistent and confident, whereas pessimists are more hesitant and doubtful.

Finally, the differences between the “optimistic” and “pessimistic” animals could be attributed to differences in their sensitivities to positive and negative feedback in the task. For example, the “optimists” could have been more sensitive to positive feedback (high reward) and/or less sensitive to negative feedback (lack of reward), and these differences could potentially be the most pronounced at the highest level of risk, when the probability of obtaining a large reward is the lowest. Further studies (e.g., using the probabilistic reversal learning paradigm; Bari et al., 2010) should provide additional insights into this issue by enabling detailed analysis of the differences in sensitivity to true and misleading positive and negative feedback in “optimistic” and “pessimistic” animals.

The present data also provided novel insights into the interrelations among risk-based decision making, cognitive judgment bias and the sensitivity of the DA system. The animals classified as “optimistic” following APO challenge displayed more stereotyped gnawing behavior than the “pessimists”, which suggests higher reactivity of the DA system in the “optimistic” animals. APO, which is a full agonist of D1 and D2 DA receptors and has similar intrinsic activity as DA, is often used to study the reactivity of the dopaminergic system (Havemann et al., 1986; Cools et al., 1993; Surmann and Havemann-Reinecke, 1995; Germeyer et al., 2002). The resulting stereotyped motility patterns are dose dependent; low doses of APO induce sniffing behaviors, which then progress to licking and gnawing behaviors with increasing doses. In addition to dose dependency, Havemann et al. (1986) observed individual differences in the stereotyped responses. An APO dose of 2 mg/kg s.c. was found to result in at least two different behavioral phenotypes in rats (Havemann et al., 1986; Surmann and Havemann-Reinecke, 1995) with respect to oral stereotyped behavior and locomotor activity. Some rats exhibited stereotyped sniffing with markedly enhanced locomotor activity, while others displayed stereotyped licking and/or gnawing with only slightly enhanced locomotor activity. Similar observations have been reported by Costall and Naylor (1973), who have suggested that compared to sniffing or locomotor activation, licking and gnawing are signs of a greater degree of general DA activation in the central nervous system after systemic administration of APO. In the same study, the authors assigned a higher score for stereotyped licking or gnawing than for stereotyped sniffing. In two strains of rats, Cools et al. (1993) and Rots et al. (1996) showed a similar pharmacological selection based on susceptibility to APO. As reviewed by Ellenbroek and Cools (2002), APO susceptible (gnawing) rats have increased levels of mRNA for tyrosine hydroxylase in the nigrostriatal and the tuberoinfundibular system, have increased levels of [125I] iodosulpiride binding and of mRNA for D1 receptors in the neostriatum, and have lower levels of noradrenaline immunoreactivity in the nucleus accumbens (NAc). As proposed by Joyce and Iversen (1984) and Havemann et al. (1986), the individual differences in stereotyped responses after administration of APO might be explained either by an incompatibility hypothesis (incompatibility between two patterns of behavior) or by competition between the nigrostriatal and the mesolimbic DA systems, for motor output pathways. Clearly, further studies, using microdialysis or *in vivo* voltammetry will help to directly pinpoint dopaminergic loci for the observed differences between “optimistic” and “pessimistic” animals.

Although interrelations among cognitive judgment bias, risk taking and DA reactivity have been demonstrated for the first time here, they are not surprising. DA is a key neuromodulator in reward-learning and reward-seeking behaviors in humans and nonhuman animals (Schultz, 2001; Wise, 2004; Belin and Everitt, 2008), and it has been shown to play an important role in behavioral guidance (Guitart-Masip et al., 2014) via value/action learning (Bayer and Glimcher, 2005) and action invigoration (Salamone et al., 1994; Parkinson et al., 2002; Satoh et al., 2003; Niv et al., 2007), as well as in maintaining the balance between different types of behavioral control (Hitchcott et al., 2007). Indeed, DA neurotransmission in the ventral striatum has been recently reported to refine cost–benefit evaluations that require risk–reward judgments (Stopper et al., 2013). In Stopper’s study, D1 receptor blockade caused risk aversion, while D1 stimulation led to optimized decision making, suggesting that a normal level of NAc D1 activity modifies decision-making biases away from or towards larger uncertain rewards to maximize the overall amount of reward that can be obtained. These effects were postulated to be mediated partially by fluctuations in tonic DA in the NAc, which appear to represent the integration of multiple types of information relevant to decision making, including reward uncertainty, opportunities to select preferred rewards, overt choice behavior, and changes in reward availability (St. Onge et al., 2012). However, in the same study, NAc D2 receptor activity was not observed to make a discernible contribution to these functions, whereas excessive D3 receptor activity blunted the impacts of larger rewards on decision biases. Notably, drugs that enhance dopaminergic function, such as L-DOPA, have also been shown to enhance a signal in striatum that represents reward-prediction errors during instrumental learning. Such drugs, thus increase the likelihood that stimuli associated with greater monetary gains will be chosen (Pessiglione et al., 2006), enhance estimates of hedonic pleasure during the imaginative construction of positive future life events (Sharot et al., 2009), and enhance optimism bias by impairing the ability to refine predictions in response to undesirable information about the future, as demonstrated in a recent study by Sharot et al. (2012).

Using an operant PD paradigm and multiple consecutive ACI tests, we have demonstrated for the first time that cognitive judgment bias is linked to risky decision making in an animal model. Additionally, using the APO sensitivity test, we attributed the observed differences between “optimists” and “pessimists”, at least in part, to differences in the reactivity of the DA system. Considering these results together with those of the human studies and our previous reports, it is apparent that “optimism” and “pessimism” exert powerful and differential effects on certain forms of cost/benefit decision making. These findings may be useful for studying cognitive deficits associated with psychiatric disorders such as depression, mania or pathological gambling.

## Author Contributions

Conceived and designed the experiments: RD, PEC, JRP and RR. Performed the experiments: RD and MR. Provided materials and analysis tools: RR, PEC and JRP. Wrote the manuscript: RR. Read the manuscript and provided critical feedback: RD, PEC, MR, JRP and RR.

## Conflict of Interest Statement

The authors declare that the research was conducted in the absence of any commercial or financial relationships that could be construed as a potential conflict of interest.
